# Clinical Report on Cases Observed at the New Orleans Charity Hospital, 1858-9

**Published:** 1859-05

**Authors:** Austin Flint


### BUFFALO MEDICAL JOURNAL

AND

### MONTHLY REVIEW.

**VOL. 14.** MAY, 1859. **NO. 12.**

### ORIGINAL COMMUNICATIONS.

ART. I.—*Clinical Report on cases observed at the New Orleans Charity Hospital*, 1858-9. By Austin Flint, M. D.

PNEUMONIA.

Under the above caption, in accordance with the wishes of the editors of the *Medical News & Hospital Gazette*, I propose to give in successive numbers of this journal, some account of my clinical observations at the Charity Hospital during the college session for 1858-9. My service at the hospital commenced on Nov. 27th, 1858, and continued for three months. It embraced two male wards, each containing fifteen beds. It rarely happened that there was a vacant bed in either ward, and, not unfrequently, temporary accommodations were made for an additional number of patients on the floor. The average number of cases under daily observation, therefore, was somewhat over thirty. The majority of the patients were affected with acute diseases, including in this category, the periodical and continued fevers. Patients were always discharged as soon after convalescence as their condition rendered it safe and proper for them to leave the hospital. Chronic cases, in many instances, did not remain long under observation, the patients sometimes prefering to leave, and sometimes being advised to do so, the prospect of improvement being considered to be better without than within the hospital wards. The service, therefore, in proportion to the number of beds, was quite active, the daily admissions ranging from four to eight daily.

It may not be amiss to state the plan of clinical instruction which was pursued. The regular daily visit was at 8 o'clock, A. M. The wards of this hospital being free, at all times, to any who choose to frequent them for clinical study, medical students and practitioners who were so disposed, attended at the morning visit, witnessing the examination of newly received patients, with reference to the diagnosis, and observing from day to day, the symptoms incident to the course of disease, together with the therapeutical measures employed in all the cases. Clinical remarks were made at the bed side; but on Wednesdays and Saturdays, after the morning visit, a clinical lecture was given in the college amphitheatre, on the cases under observation in the hospital wards. At these lectures the diseased organs, in fatal cases, were exhibited to the class. During the three months service, the more important of the diseases which occur in general practice were studied at the bedside, and discussed in the lecture room.

Daily records were made of all the important cases. The greater part of this labor was performed by myself, but I was aided here, as well as in other duties connected with the service, by my clinical assistant, Mr. Gustavus Devron, to whom I am under much obligation for his assiduity and fidelity; and I avail myself also of this opportunity of expressing my acknowledgments to Messrs. Holt, Mercer, Smyth, Stone, and other of the resident students of the hospital, for valuable assistance in my clinical pursuits.

I have selected, as the subject of this paper, the cases of *pneumonia* received into my wards during my term of service. The number of cases observed is fifteen. Full notes were taken of all these cases. I shall give a condensed summary of the history of each case, presenting some of the more important points, and omitting details which are unimportant to my present purpose, which is, not to contribute material toward extending our knowledge of the symptomatology of pneumonia, but to select from the notes of the cases, respectively, sufficient of the signs and symptomatic phenomena to show that the diagnosis was correctly made, and to give a general idea of the severity of the disease in the individual cases, its association in some instances with other affections, and to specify the remedies employed in each case.

After giving, thus succinctly, the histories, I shall offer certain conclusions and general remarks based on the clinical study of these cases. In sketching the histories, I shall enumerate the cases in the order of their occurrence. I may mention, in advance, that all the cases actually observed ended in recovery. A single case of pneumonia, only, ended fatally, and in this instance the patient was admitted *in extremis*, and died before my visit on the following morning. This case, in reality, did not come under my observation prior to the autopsy.

CASE I.—*Pneumonia affecting lower lobe of left lung. Seventeen days in Hospital.*

Thos. Corrigan, aged 37, Irish, laborer, admitted Nov. 18th. Had intermittent fever last summer. Confined to bed two weeks before admission.

*Physical signs*: Marked dullness on percussion over lower lobe of left lung. Respiration over this lobe bronchial and broncho-vesicular. Bronchophony, and the bronchial whisper intense and acute over this lobe. True crepitant and the sub-crepitant râles. Friction found over upper lobe of left lung. Over this lobe vesiculo-tympanatic percussion-sound.

No febrile movement; respirations 20.

*Treatment.* Sulphate of quinia, grs. v, three times; brandy, ℥ii, three times, and nutritious diet. The quinia apparently caused vomiting, and was suspended on the second day, the sulphate of morphia, gr. $\frac{1}{4}$, three times, being substituted. The morphia was continued for three days only, and the subsequent treatment consisted solely of brandy and nourishment.

At the time of his discharge, the disparity between the two sides, as regards physical signs, was very slight.

CASE II.—*Pneumonia affecting lower lobe of right lung, complicating Typhoid fever. Twenty-two days in Hospital.*

Peter Boyle, aged 25, admitted 17th November. Had taken to the bed two days before his admission. He presented the symptoms diagnostic of typhoid fever; the eruption appeared on the 25th of November. On the 26th, the physical signs denoted pneumonia affecting the lower lobe of the right lung. The disparity between the two sides, as regards the resonance on percussion was marked. The auscultatory signs of solidification, viz: bronchial respiration, bronchophony, and the bronchial whisper, were not strongly marked. The grade of the typhoid fever was mild. The treatment consisted of small doses of the sulphate of morphia to allay cough; milk punch and nutritious diet.

The patient was discharged quite well, Dec. 9th.

Case III. *Pneumonia, affecting lower lobe of right lung. Nine days in hospital.*

Louis Cussy, Frenchman, aged 42, admitted Nov. 20. Took to the bed the day before his admission, but had had cough for a week. Three weeks before his admission he had intermittent fever, at first of the quotidian, and afterward of the tertian type. The last paroxysm was twelve days before his admission, The expectoration was small, adhesive, rusty; pulse 92; respirations 20. Circumscribed flush of right check.

*Physical Signs.* Notable dullness on percussion on the right side, bounded by a line coincident with the lower interlobar fissure. Bronchial respiration wanting. Crepitant râle. Soft, rubbing friction-sound below line of interlobar fissure in front and behind. Well marked bronchial respiration, bronchophony and the bronchial whisper were not, at any time, marked in this case.

*Treatment.* The sulphate of quinia, gr. v, three times daily, was contiu ued for four days; and the sulphate of morphia, gr. $\frac{1}{4}$, three times daily. This, with brandy ℥ii, three times daily, and nutritious diet, constituted the treatment.

When discharged, Dec. 1st, the respiratory murmur was developed and vesicular over the affected lung, but an obvious disparity on percussion continued.

The patient, as I afterward learned, was quite intemperate, and he was discharged, although still weak, at his urgent request.

Case IV. *Pneumonia affecting the upper lobe of the left lung, in a patient recently discharged convalescing from pneumonia affecting the lower lobe of the right lung,* (*Case* 3.) *Death fifteen hours a,ter admission.*

Louis Cussy, the some patient as in No. 3, was admitted Dec. 13th, thirteen days after his discharge, with pneumonia affecting the upper lobe of the left lung, and died during the night. The patient was not seen by me, but he was examined by Dr. Alston, of Texas, and other members of my private class, and the existence and seat of the disease determined by the physical signs.

On examination after death the upper lobe cf the left lung was enlarged in volume and completely solidified; inelastic, non-crepitant, undergoing no change on inflation, sinking in water, anæmic, granular—in short, presenting all the anatomical characters distinctive of the second stage of pneumonia, with slight exudation of lymph on the pleural surface.

The lower lobe of the left lung was œdematous, serous liquid, scarcely spumous, escaping in abundance on section, but no solid or granular deposit.

It was ascertained that the patient, after his discharge, had indulged in excessive drinking. On the day of his death he walked three miles to enter the hospital.

This case illustrates the recurrence of pneumonia in another lobe, and, in fact, the opposite lung; not in the portion previously affected, which it might be supposed, *à priori*, having been so recently affected, would be thereby rendered liable to a renewed attack of inflammation. The patient was affected with two distinct attacks of pneumonia, there being no pathological connection between the two attacks other than that both probably involved the same internal and external causative conditions.

CASE V. *Pneumonia affecting the lower lobe of right lung. Eight days in hospital.*

John Martin, aged 30, Irishman, laborer, admitted Nov. 22d. Patient accustomed to drink rather freely. Had been attacked eight days before with chill, pain in right side, cough and bloody expectoration. Took at once to the bed, but after the first day sat up a portion of the time.

Cough and expectoration, at the time of his admission, slight, the matter expectorated being muco-purulent. No febrile movement, and the respirations 20. Slight circumscribed flush of right cheek.

*Physical Signs.* Marked dullness on percussion within the limits of lower lobe of the right lung. Over the upper lobe of the right lung the percussion sound vesiculo-tympanitic. Crepitant râle. Respiratory sound over the affected lung feeble and broncho-vesicular. Well marked bronchial respiration not present. Bronchophony also wanting. Tolerably intense and acute bronchial whisper.

*Treatment.* The patient having formerly had intermittent fever, the sulphate of quinia, gr. v, three times daily, was continued for several days, with brandy, ℥ii, three times daily, and nutritious diet. This constituted the treatment, with the exception of castor oil on the day before his discharge.

Discharged quite well, Nov. 29th.

Case VI. *Pneumonia affecting the lower lobe of the right lung. Complicating Typhoid Fever of severe grade. Slow resolution of pneumonic solidification.*

Henry Corcoran, Irishman, laborer, aged 28, admitted Nov. 24th.

He had taken to the bed four days before his admission, having, at that time, lancinating pain referred to the right lateral surface of the chest, with cough.

At the time of his admission he had cough with adhesive, semi-transparent and slightly rusty expectoration.

The *physical signs* were, marked dullness on percussion over the lower lobe of the right lung; crepital râle; a feeble, acute bronchial whisper. Well marked bronchial respiration and bronchophony were not developed in this case.

The patient presented all the diagnostic characters of typhoid fever, inclusive of the eruption. The latter affection was of a low and severe grade; the pulse frequent and feeble; muttering delirium with efforts to get out of bed; dejections in bed; carphologia, subsultus, etc.

The *treatment* consisted of the free administration of alcoholic stimulants, essence of beef and milk for diet, and small doses of the sulphate of morphia. The patient was convalescent, Dec. 4th, fourteen days from the time of taking to the bed; the convalescence, however, was slow, being retarded by sloughing over the hips and sacrum.

The resolution of the pneumonia was tardy, but equality of the two sides over the lower lobe was nearly restored, as regards percussion and auscultatory signs, by Jan'y 1st.

The patient remained in hospital at the end of my service, having gained progressively in flesh and strength, but affected with dry cough, accompanied with dullness on percussion at the right summit of the chest. The latter, existing without a corresponding disparity in the auscultatory phenomena, was attributed to enlargement of the bronchial glands incident to typhoid fever.

Case VII. *Pneumonia affecting the lower lobe of the right lung. Twelve days in hospital.*

Joseph Clark, aged 27, English, laborer, admitted Nov. 24th.

He had been confined to the bed for six days prior to his admission. At the time of his admission, the expectoration was semi-transparent, adhesive and rusty. Pulse 80; respiration 20.

*Physical Signs.* Marked dullness over the lower lobe of the right lung; crepitant râle with the few first inspirations; the respiration extremely feeble, so that its character could not be satisfactorily studied; feeble but acute bronchial whisper. Over the upper lobe of the right lung, the percussion-note sonorous and vesiculo-tympanitic.

The treatment at first consisted of opium, gr. ii, every four hours, with nutritious diet. The quantity of opium in two days was reduced to gr. i, and the sulphate of quinia, grs. ii, added. These remedies were discontinued six days before the patient was discharged.

When discharged, Dec. 6th, slight disparity on percussion existed over the lower lobe, but all pulmonary symptoms had ceased.

CASE VIII. *Pneumonia affecting the lower lobe of the right lung. Fifteen days in hospital.*

George Nelson, aged 24, American, admitted Nov. 29th. Had had cough with pain in the side for three days, but did not take to the bed prior to his admission.

The expectoration was adhesive and muco-purulent. From his description it had been rusty before his admission. Pulse 76; respiration 28.

*Physical Signs.* Marked dullness on percussion over the lower lobe of right lung; crepitant râle, abundant and diffused; bronchophony and bronchial whisper acute and tolerably intense. Well marked bronchial respiration not developed in this case.

The treatment consisted of opium, grs. ii, three times daily, and nutritious diet. This was continued for seven days. No medicine afterward.

The patient was discharged, quite well, Dec. 14th.

CASE IX. *Pneumonia affecting the upper lobe of the right lung. Thirteen days in hospital.*

Michael Henney, aged 35, laborer, admitted Dec. 1st. He had been ill for two and a-half months, and so far as the previous history could be obtained, taking into view the present signs, it appeared that he had had intermittent fever, and pneumonia affecting the lower lobe of the right lung.

The *physical signs* denoted consolidation of the upper lobe of the right lung. Marked tympanitic, i. e., non-vesicular sonorousness existed in front above the fourth rib and in the upper scapular space behind. Crepitant râle in the latter situation and sub-crepitant râle in front. Bronchial respiration not well marked, but bronchophony tolerably strong, and the bronchial whisper intense and acute. Relative dullness and a feeble vesicular murmur over the lower lobe of the right lung.

The expectoration quite copious, a portion muco-purulent, and the remainder semi-transparent, adhesive and rusty. Pulse 88; respirations 20.

*Treatment.* Sulphate of quinia, grs. v, and opium, gr. i, three times daily. The opium was afterward increased to grs. ii, three times. On the fifth day, the quinia was discontinued, and the sulphate of morphia given in doses of gr. $\frac{1}{4}$, three times daily, with brandy, ℥vi, three times, and nutritious diet.

On the ninth day medicine was discontinued, full diet continued, and on the thirteenth day (Dec. 13th) the patient was discharged, slight dullness remaining over the upper lobe of the right lung, but the respiratory murmur well developed and vesicular.

CASE X. *Pneumonia affecting the lower lobe of the left lung; the inflammation subacute. Six days in hospital.*

Lorentz Egert, German, aged 45, laborer, admitted Dec. 7th. Had been ill three days before his admission. The symptoms belonging [illegible] the previous history are not noted.

He presented marked circumscribed flush of both cheeks; cough and expectoration slight, and the characters of the latter not observed; respirations 16, and pulse 74.

*Physical Signs.* Well marked dullness on percussion over the lower lobe of the left lung. Distinct and abundant crepitant râle. Bronchial respiration wanting. Vocal resonance greater over the lower lobe of the left than right lung; moderately intense and acute bronchial whisper.

*Treatment.* Opium, grs. ii, three times daily, and nutritious diet.

The patient was discharged on Dec. 13th, being free from cough; slight relative dullness remaining over the affected lobe, the respiratory murmur feeble, but vesicular, with an occasional sub-crepitant râle.

CASE XI. *Pneumonia affecting the upper lobe of the right lung. Twelve days in hospital.*

John Lavell, aged 40, Irish, laborer, admitted Dec. 14th. The patient had not been well for ten months before his admission, having been affected with intermittent fever. The date of the existing pneumonia does not appear in the record of the previous history.

The expectoration at the time of his admission, was abundant, semi-transparent. adhesive, and had a yellow tint. Pulse 80; respirations 22.

*Physical Signs.* Notable dullness with tympanitic quality of sound on percussion on the right side extending in front from the summit to the lower margin of the third rib, and in the upper scapular space behind. Over the lower lobe of the right lung the sonorousness greater than on the left side, and the quality vesiculo-tympanitic. Well marked bronchial respiration over the upper lobe of the right lung in front and behind, with bronchophony and an acute bronchial whisper. The respiratory murmur over the lower lobe of the right lung more intense than on the left side and equally vesicular.

*Treatment.* Sulphate of quinia, grs. v, and opium, grs. ii, three times daily, were continued for five days. The opium was then discontinued, and the quinia continued in doses of three grains, three times daily, with milk punch and full diet.

The patient was discharged, Dec. 56th, reporting quite well, relative dullness existing at the summit of the chest on the right side, but the respiratory murmur well evolved and vesicular, the bronchophony and acute bronchial whisper having disappeared.

CASE XII. *Pneumonia affecting the lower lobe of the left lung. The patient tuberculous. The pneumonia developed in hospital.*

Bryan Campbell, aged 20, Irish, gardner, admitted Dec. 20th.

Cough had existed for two years, and he had had repeated attacks of hæmoptysis. The physical signs denoted a considerable deposit of tubercle at the summit on the right side.

Eight days after his admission the expectoration became adhesive and hæmorrhagic. Respirations 30; pulse 115.

*Physical Signs.* Marked dullness over the lower lobe of the left lung. Crepitant râle. Weak bronchial respiration and bronchophony over this lobe

January 27th, the dullness and auscultatory signs of solidification of the. lower lobe of the left lung had disappeared. The signs of tuberculous solidification at the summit on the right side continue.

*Treatment.* Prior to the development of the intercurrent pneumonia, the patient was placed on the hypophosphite of soda, ℈i, three times daily, small doses of the sulphate of morphia, milk punch and full diet. No change in the treatment was made during the continuance of the pneumonia.

After recovering from the pneumonia, the patient desired to leave the hospital and was discharged. In a few days, however, he returned, finding that he had not breath enough to work, and he remained in hospital when my term of service ended, his condition being about the same as before the attack of pneumonia.

CASE XIII. *Pneumonia affecting the lower lobe of the left lung. Delirium tremens. Sixteen days in hospital.*

William Coughlan, aged 29, Irish, laborer, admitted Dec. 26th. He was attacked with cough, etc., on the 22d, and took to the bed on the 23d.

Expectoration semi-transparent, adhesive and rusty. The pulse 120; respirations 40. Lancinating pain referred to the lower anterior portion of the left side of the chest.

*Physical Signs.* Notable dullness over the lower lobe of the left lung. Feeble crepitant râle. Bronchial respiration well marked, with intense bronchophony and an acute bronchial whisper. Percussion-sound vesiculo-tympanitic over the upper lobe of the left lung.

*Treatment.* Opium, grs. ii, three times daily. On the 28th the opium was increased to grs. iii, every six hours. On the 29th the pulse was 100, and the respirations 24. The treatment of the 28th was continued, with milk punch and the essence of beef and milk for diet.

Delirium was manifested on the 29th. It increased on the 30th, and presented the characters of delirium tremens. During the night of this date and the succeeding night, he was strapped to the bed by order of the captain of the night watch, as is usual in this hospital in such cases. The pulse fell to 80 and the respirations to 20. The physical signs denoted diminished solidification of the affected lung. The treatment now consisted of the sulphate of morphia, gr. ss, every four hours, and brandy, ℥ii, hourly, with essence of beef for diet.

Sleep occurred on January 1st. The brandy and morphia were continued in diminished doses.

Jan'y 3d, the patient was convalescent, the bronchial respiration and bronchophony having disappeared.

Jan'y 11th he was discharged, quite well.

Case XIV. *Pneumonia, affecting the lower lobe of the left lung. Delirium tremens. Ten days in hospital.*

Nicholson, aged 27, boatman, admitted Jan'y 25.

He was attacked suddenly with pain in the side and cough, on the 18th of Jan'y, and at once kept the bed. He continued to drink spirits freely after the attack up to the time of his admission.

Delirium was manifested on the second night after his admission. It assumed the character of delirium tremens the day following.

Expectoration small, adhesive, and deeply rusty. Pulse 100; respirations 28.

*Physical Signs.* Notable dullness over the lower lobe of the left lung Intense bronchial respiration; marked bronchophony and the bronchial whisper intense and acute. Vesiculo-tympanitic resonance over the upper lobe of the left lung. Crepitant râle.

*Treatment.* At first, opium, grs. ii, three times daily, and brandy, ℥ii, every three hours. The third day, the sulphate of morphia, gr. ss, every four hours, and brandy, ℥ii, every two hours, with essence of beef for diet. On the fourth day the delirium continued, and the sulphate of morphia, in doses of gr. i, was prescribed, and brandy, ℥ii, every two hours, with essence of beef. Six grains of the morphia were given between 9, A. M. and 11, P. M. He was strapped to the bed all night by direction of the captain of the night watch. During the night, brandy, ℥ii, hourly, was given. He slept during the latter part of the night, and was quiet in the morning, taking food with relish. Pulse 100; respirations 16.

The morphia and brandy were continued in small doses, and he convalesced immediately.

The bronchial respiration and bronchophony were absent on examining the chest after the delirium ceased, showing the rapid resolution of the lung notwithstanding the delirium.

Medicine was discontinued on Jan'y 31st, and the patient was discharged, quite well, Feb'y 4th.

CASE XV. *Pneumonia, affecting the lower lobe of the left lung, with an abundant liquid effusion into the pleural sac.*

William O'Neil, aged 24, Irish, laborer, admitted January 27th. He was attacked suddenly the day before his admission, with acute pain in the side. Slight cough had existed for two days prior to the attack.

The acute pain continued at the time of his admission, referred to the neighborhood of the nipple, and extending to the base of the chest, in front, on the left side. Expectoration small, rusty, and adhesive. Respirations 50, the inspiration abruptly arrested on account of pain. Pulse 100.

*Physical Signs.* The right side presenting the superior and inferior costal movements marked, while the left side is nearly motionless. Clear percussion resonance on both sides in front; the respiratory murmur intense on the right, and quite feeble on the left side. Marked dullness on percussion over the lower lobe of the left lung, and a feeble crepitant râle. The respiratory sound not sufficiently developed over this lobe to study its characters. Weak bronchophony over this lobe and an acute bronchial whisper.

The day following, the acute pain had ceased. Marked dullness over the lower lobe of the left lung continued, and feeble bronchial respiration was discovered. In front dullness on percussion, extended upward above the interlobar fissure to the third rib, in the sitting posture, and variation of the upper limit of this dullness with change in the position of the patient was observed. Tympanitic sonorousness above the level of the liquid. Pulse 96; respirations 28.

*Treatment.* Opium, grs. ii, every four hours.

Jan'y 30th. The physical signs remained the same, and the treatment was continued, brandy, ℥ii, every four hours being added, with essence of beef for diet.

Jan'y 31. The level of the liquid lowered an inch, the patient in a sitting posture. The bronchial respiration and bronchophony more marked over the lower lobe of the left lung. Percussion-resonance, vocal-resonance, the respiratory murmur and vocal fremitus all showed the expansion of the upper lobe as low as the nipple. Pulse 88; respirations 24.

Treatment continued.

Feb'y 1st. The signs of expanded lung extend to the situation of the interlobar fissure in front. Bronchial respiration and bronchophony continue over the lower lobe of the left lung.

Same treatment.

Feb'y 2d. The bronchial had given place to the broncho-vesicular respiration, and the bronchophony to increased vocal resonance, over the lower lobe of the left lung. Cough and expectoration slight. Pulse 56. No dejection for a week.

Treatment: castor oil.

From this date the patient could be regarded as convalescent. He sat up a little on the day following. The physical signs denoted progressive and rapid resolution of the consolidation of the inflamed lobe; the cough and expectoration ceased, but the patient complained of soreness of the left side, for which a belladonna plaster was prescribed. At the end of my term of service, Feb. 13th, he had been designated to stand on watch four hours during the night, and was about ready to be discharged.

The rapid removal of the effused liquid, as well as the solid exudation, under the treatment pursued, is to be noted in this case.

Lest the reader may infer that the brief account which has been given of the foregoing cases represents the extent of the records at the bedside, I repeat that my present object, aside from the treatment, is simply to introduce details to constitute sufficient evidence of the diagnosis (assuming accuracy on the part of the observer) and to give a general idea of the severity of the individual cases derived from the intrinsic intensity of the disease, and association with other affections.

### *Conclusions and Remarks.*

Of the fifteen cases, in all the pneumonia was limited to a single lobe. In one instance two lobes were successively attacked, (cases 3 and 4) recovery from the first attack having taken place before the second occurred. This was probably true in another instance, (case 9). The lower lobe was the seat of the disease in all save two cases. In one of these, (case 4,) the lower lobe had been previously affected, and this was probably true of the other instance, (case 9).

Of the thirteen cases in which the lower lobe was affected, the disease was seated in the right side in seven, and in the left side in six.

The pneumonia was primary in twelve of the fifteen cases. Of the three instances in which it was developed secondarily, it occurred as a complication of typhoid fever in two, and as an intercurrent affection in pulmonary tuberculosis in one instance.

In the twelve primary cases, the disease was uncomplicated in all but three instances. Delirium tremens was developed as a complication in ten cases; and in one instance, pleuritis with considerable liquid effusion coëxisted. More or less pleuritis is almost uniformly present in cases of pneumonia, but it is certainly rare for the pleuritis, under these circumstances, to be attended by much effusion.

The ages of the patients varied between 20 and 45, the mean age being about 30. In nearly every instance the patients were robust, and in good health when attacked with the disease. Several were intemperate, and several were subject to attacks of intermittent fever.

Exclusive of three cases remaining in hospital at the end of my term of service (two of these remaining with other affections), and of the case which ended fatally, the length of stay in hospital varied from six to twenty-two days; the mean period being a fraction over thirteen days.

A fatal result occurred in one case only. In this case, as already stated, the patient had been discharged a few days before, convalescent from an attack of pneumonia affecting the lower lobe of the right lung. He resumed his habits of intemperance directly he was discharged, and was seized a second time with pneumonia, now affecting the upper lobe of the left lung. He walked to the hospital, a distance of three miles, after being attacked, and died by asthenia, fifteen hours after his admission, without coming under my observation.

Convalescence was rapid in all instances, save one in which the pneumonia occurred as a complication of typhoid fever. Exclusive of this instance, the resolution of the affected lung, as shown by the disappearance of the physical signs denoting solidification, was rapid. The resolution was in all instances complete, slight relative dullness on percussion only remaining, which, as is well known, persists for some time after recovery from pneumonia. In all instances the symptoms of the disease, viz., cough, expectoration, etc., completely disappeared. In no instance were the patients left enfeebled, but at the time of discharge they all reported themselves able to return to their avocations, and the majority were day laborers.

The treatment pursued in these cases, as the reader has doubtless remarked, was not complex. The sulphate of quinia was given, in tolerably full doses, in the cases in which intermittent fever had previously existed. This remedy was not prescribed with special reference to the pneumonia, but in order to forestall the development of intermittent fever as a complication, Quinia has been extolled as a valuable remedy in pneumonia, irrespective of the liability of the disease to become complicated with intermittent fever if the patient reside in a malarious section, and, especially, if he has been subject to attacks of the latter affection. However this may be, it is certain that the remedy, given in full doses, does not exert an unfavorable influence on the progress of the pneumonia. It is not contra-indicated by this affection. To prevent the development of intermittont fever, and, if developed, to arrest the paroxysms as speedily as possible, are important objects of treatment in certain cases of the disease under consideration. The combination of the two affections often places the patient in imminent danger, when with either affection, singly, his condition would not be serious. Happily, under these circumstances, we possess a special remedy by which we may expect to control one of the affections. This, then, is the leading indication, and the life of the patient may depend on its being promptly and effectively fulfilled. The practitioner who gives precedence to indications derived from the pneumonia, under these circumstances, or who so divides his attention as to fulfill incompletely the leading indication, commits an error which may be fatal to the patient. As a rule, it is well to recognise, at the commencement, a liability to this complication, and to forestall its occurrence, especially when the protective remedy, to say the least, does not interfere with the favorable progress of the pneumonia.

Opium entered more or less into the treatment of these cases. My observations have led me to regard this as a valuable remedy in pneumonia. As a palliative of pain, it fulfills an important indication; for pain, in addition to the suffering which it occasions, determines an afflux of blood to the painful part. This fact is illustrated in neuralgia affecting the supra orbital nerve. During the paroxysm of pain, the eye becomes injected, and the redness rapidly disappears after the pain has been relieved by an opiate. But there is reason to believe that opium exerts a salutary effect beyond the relief of pain. I have repeatedly noted marked diminution in the frequency of the pulse and respirations, in cases of pneumonia, a few hours after the patient had taken full doses of opium. It appears to lessen the perturbatory effects in the economy of the local inflammation, if, indeed, it does not diminish the intensity of the inflammatory action. Some practitioners are deterred from the use of this remedy in pneumonia, by the idea that it interferes with the removal, by expectoration, of the exuded products of inflammation. But how often do we observe the rapid disappearance of the solidification in pneumonia, with slight expectoration, or none whatever! The intra-vesicular exudation is, for the most part, absorbed, not expectorated, a fact not strange when the structure of the cells, and the facility of endosmosis in this situation are considered. Interference with expectoration, therefore, if true with respect to the employment of opium in pneumonia, is not a valid objection. The merits of the remedy, however, in this application, must rest on clinical experience; and I cannot but think that this paper will be of some utility, should it serve to remove from the minds of some readers, groundless apprehensions with respect to the free use of opium in pneumonia.

Alcoholic stimulants were employed, to a greater or less extent, in the treatment of these cases, and in all the cases the patients were placed on a nutritious diet. The abstract notion that stimulants and sustaining food are inconsistent with the treatment due to local inflammation, is a remnant of Broussaism which still exerts considerable influence on medical practice. It suffices to destroy this notion, to bear in mind the significant injunction of Chomel, viz., *not to treat diseases, but to treat patients affected with disease.* With regard to stimulants, it may be stated, as a rule, that they are indicated in cases of local inflammation, whenever the patient in health is addicted to their use. They cannot with safety be withheld, under these circumstances, if the local inflammation involve, from its seat or intensity, danger to life. But without reference to previous habits, the use of stimulants in the treatment of pneumonia, and of other local inflammations, is important in proportion as it becomes an indication to support the powers of the system. It is, perhaps, an error in medical practice as common and as serious as any, to overlook or depreciate this indication in the management of local diseases. Practically, the existence of this indication and its urgency, in individual cases, may be brought clearly and forcibly before the mind by proposing, at the bedside, the following questions: Is the patient in danger of death? If death occur will it take place by asthenia? How imminent is the danger of death by asthenia? Whenever, in the management of disease, the physician has reason to fear that he may lose his patient by asthenia, and in proportion to the tendency to a fatal result by that mode of dying, alcoholic stimulants, as a rule, form an important part of the measures indicated, on the same ground that they enter into the treatment of fevers. And this remark applies equally to nutritious diet, viz., the animal essences, etc. Cases of pneumonia occur in which patients are to be saved by pursuing, boldly and perseveringly, the supporting treatment precisely as in cases of low typhus or typhoid fever. And the indications for this plan of treatment may be present early, as well as late in the progress of the disease

The union of delirium tremens, with pneumonia, for example, often places the patient in imminent danger of death by asthenia. The free use of stimulants and concentrated nutriment, is requisite to carry him safely through the perils of this combination. Two of the cases included in the present collection, (Nos. 13 and 14,) illustrate this remark.

But even when the danger to life is not great, it is an object to support the system with a view to a speedy and rapid resolution of the inflammation. Alcoholic stimulants may not be required for this object, but it will be promoted by a nutritious diet. The fear of feeding the disease by feeding the patient, is a vulgar notion not warranted by clinical observation. I do not hesitate to allow patients to take food as nutritious in quality and as freely as they desire. The late Dr. Graves was so impressed with the importance of supplying the system with nourishment in febrile diseases, that he desired to be placed as an epitaph on his tomb, "he fed fevers"! He might have extended the circle of diseases in which feeding is important. It is hardly less important, in some instances, speaking metaphorically, to feed pneumonias, than to feed fevers, and, indeed, feeding, as an essential element of supporting treatment, is important in any disease when it is an indication to obviate the tendency to death by asthenia.

With regard to the time when an active supporting plan of treatment, (stimulants and concentrated nutriment) is indicated, and the extent to which it is to be carried, I will make but a single additional remark. If there be room for doubt on these points, it is better to err by commencing too soon, and pushing it too far, than by delay and insufficiency; for while it is easy to discontiuue or diminish supporting measures before much if any actual harm is done, time which has been lost cannot be recovered.

Quinia, opium, alcoholic stimulants and nutritious diet, constituted the treatment in the cases embraced in this report. The grounds for the non-employment of certain therapeutical measures, in these cases, are now to be conisdered, Blood-letting, general and local, tartar emetic, the veratrum viride, mercury, cathartics, blisters and other modes of counter-irritation are employed, to a greater or less extent, in the treatment of pneumonia. None of these remedies, however, entered into the treatment of these cases. This fact, without explanation, might lead to erroneous inferences as regards the views of the writer.

Blood-letting in pneumonia has, of late, been much discussed. I have contributed my mite toward this discussion,* and do not propose to enter

* Report on Blood-letting in Pneumonia, Buffalo Medical Journal, Vol. xi. 1856. Page 288.

upon it in this paper. I will simply remark that, in my opinion, it is an error to assume blood-letting to be never useful in pneumonia, albeit it is a far greater error to advocate the indiscriminate employment of this spoliative measure in that affection. The propriety of blood-letting, however, is rarely a question in hospital cases of pneumonia. In the great majority of instances when patients are admitted into a hospital, the disease has advanced to the second stage; one or more lobes are solidified, and, under these circumstances, the abstraction of blood will, in general, only tend to retard resolution. Had these cases been under observation from the commencement of the disease, it is possible that blood-letting might have been indicated in some instances, not with a view to arrest the inflammatory action, nor to limit its extent, nor to lessen the amount of exuded products; but to diminish the intensity of symptomatic febrile movement, relieving the heart of over accumulation of blood in its cavities, and consequent over tasking of its powers. With this limited view of blood-letting, making due allowance for its evils, it is indicated in only a small proportion even of the cases of pneumonia which come under observation at the onset of the disease.

The tartar emetic and veratrum viride undoubtedly control, in a marked degree, the frequency of the heart's action, and, so far, diminish, for the time, symptomatic febrile movement. It remains, however, to be determined, to what extent this effect is important with reference to the inflammatory process and resolution. We can at this day understand how Laennec was deceived in attributing the rapid removal of the exudation in pneumonia to the influence of tartar emetic; for it had not then been observed, as it has been since his day, that the exudation disappears, in some instances, quite as rapidly without any treatment. These cardiac sedatives, if useful when the symptomatic febrile movement is intense, are certainly not indicated when the action of the heart is but slightly or moderately increased, as in a pretty large proportion of the cases of pneumonia in which the affection is limited to a single lobe, especially after exudation has taken place.

The action of mercury has been considered as useful in two ways in pneumonia, viz., limiting the amount of exudation and promoting its absorption. With reference to the first of these supposed effects, the exudation in pneumonia generally takes place, to its fullest extent, before mercurialization can be produced. With reference to the second effect, the exudation is absorbed, in favorable cases, quite rapidly without mercurialization. Without, therefore, presuming to deny altogether the so-called anti-plastic and the sorbefacient powers of mercury, I believe that it is a remedy generally uncalled for in the treatment of pneumonia. The occurrence of salivation at the time of convalescence, is, to say the least, an inconvenience which, if not necessary, it is desirable to avoid. Having for many years relinquished the use of this remedy with reference to its special or constitutional effects in treatingthis disease, I am satisfied that it may with safety and advantage be dispensed with.

Cathartics, as a means of depletion during the early stage of pneumonia, may be indicated. If adequate to fulfill the object of depletion, they are certainly to be preferred to blood-letting, since they do not involve an expenditure of the orgauized constituents of the blood, and are therefore not spoliative. Except for this purpose, it may fairly be doubted whether they are called for in pneumonia. They conflict with supporting measures when these are indicated. That they are not important with reference to the absorption of the exudation, is sufficiently illustrated by the cases reported in this paper. In one of the cases, (No. 15,) considerable liquid effusion, together with the solidifying deposit, disappeared in a few days, although, inadvertently, the bowels had been permitted to remain constipated for a week.

Blisters and other modes of active counter irritation, when employed in pneumonia, are, of course, considered as acting usefully by way of revulsion. I suppose that no judicious physician attributes to them this action during the first stage of the disease. To employ them in this stage is to add a certain amount of cutaneous inflammation to the existing pulmonary inflammation. After solidification has taken place, all the results to which the inflammation may be expected to give rise, have already occurred. Revulsion, then, were it is attainable, ceases to be an indication. Is it imagined that a traumatic inflammation of the skin diminishes the afflux of blood to the affected lung? But the affected lung in the second stage of pneumonia is already anæmic! The inconvenience occasioned by blisters to the patient is considerable; they are also a source of inconvenience to the practitioner, by interfering with the daily examinations of the chest in order to determine the physical condition of the affected lung. These remarks, of course, do not apply to mild revulsive applications, such as sinapisms, and to fomentations which doubtless possess a certain value in the treatment of pneumonia.

The several therapeutical measures, thus, which have heeu enumerated, as not entering into the treatment of the cases of pneumonia now reported were not employed, because, with reference to some of them, the disease did not come under observation sufficiently early to find the indications for their use, or, the indications existing in a certain proportion of cases only, they did not happen to be present in these cases; and with reference to other of the measures mentioned, their value and propriety in the disease under consideration are, to say the least, doubtful. As the modern conservative surgeon rejects the doctrine contained in the old aphorism, *melius anceps remedium quam nullum;* so the conservative physician should refuse to resort to remedies which are either unnecessary or of questionable utility, except in the cases in which a deviation from this rule is warranted for experimental observation. I cannot but think that the practice of medicine would gain much if practitioners, instead of deliberating at the bedside as to whether this or that potent remedy is called for, were oftener in the habit of propounding to themselves this inquiry: Are there present any clear indications for active interference.

The last remark suggests a point of fundamental importance in the treatment of this, as well as other acute diseases, viz., its intrinsic tendency as regards termination. Does pneumonia, in itself, tend to destroy life? We might answer this question by citing the results as reported by different practitioners, the disease being treated by different and sometimes quite opposite measures. But the clinical study of the disease uninfluenced by medication, pursued of late years on an extended scale, enables us to answer the question more directly. The results reported by Dr. Dietl, and others, in which, in a large number of cases, no active remedies were employed, show that pneumonia, limited to a single lobe, and uncomplicated, ends in recovery in the vast majority of instances. This statement is, of course, exclusive of the epidemic forms of the disease; but it is probable that the fatality of epidemic pneumonia depends generally on some important complication, or associated affection. A series of cases of sporadic, uncomplicated pneumonia, therefore, ending favorably, under a certain plan of treatment, does not afford adequate evidence that the success was due to the treatment. It would be a fairer conclusion to impute the success to the intrinsic tendency of the disease to recovery. On the other hand, a series of cases in which the fatal cases were not few, would justify, at least, a suspicion that the want of success might be due to the treatment. It would, however, be on error to suppose that because pneumonia, under favorable hygienic circumstances, generally ends in recovery without medicinal treatment, that medication is consequently never called for. Better far, indeed, no treatment than injudicious interference; but judicious treatment may nevertheless save some lives which would be lost under the expectant plan. Moreover, there are objects of treatment in disease, in addition to recovery. Relief of distressing symptoms during the progress of disease; a cure *cito et jucunde,*

as well as sure; a convalescence rapid, and a recovery complete, leaving the powers of the body not permanently impaired — these are important ends to be kept in view in medical practice. The natural history of a disease, and its intrinsic tendency to life or death, constitute the true point of departure for the study of therapeutics in relation to that disease; but medical art should not be content with being able to state the chances of recovery, even when the chances are vastly in favor of this termination.

